# Supplementary material for: Arrival and diversification of mabuyine skinks (Squamata: Scincidae) in the Neotropics based on a fossil-calibrated timetree
Source: PeerJ. 2017 Apr 18;5:e3194. doi: 10.7717/peerj.3194 (PMC5398276; doi:10.7717/peerj.3194)
Supplement: Supplemental Information 1 — GenBank GI accession numbers of sequences used in this study. [file peerj-05-3194-s001.pdf]

	12S rRNA	*16S rRNA*	*cmos*	*cytb*	*enol*	*gapdh*	*gpr149*	*myh*
Ablepharus	34821616	49476602	2760966	49476366	-	-	-	-
Acontias	33385521	33385469	334903274	238867256	-	39545954	-	82549050
Afroablepharus	34821618	34821532	158121080	158121062	-	-	-	-
Amphiglossus	38884825	255766973	38884934	38884885	40068155	39545962	392936187	-
Anomalopus	321271330	30691204	334903276	-	-	-	-	-
Aspronema cochabambae	17221682	17221687	-	-	-	-	-	-
Aspronema dorsivittatum	82548850	387166131	82548407	82548729	82549024	82548569	-	82549054
Ateuchosaurus	61102831	-	61102774	238867260	-	-	-	-
Bassiana	61102827	-	334903280	61102873	-	-	-	-
Bellatorias	-	-	334903282	-	-	-	-	-
Brachymeles	55831606	34821426	61102788	238867262	321494418	-	392936207	-
Brasiliscincus agilis	82548827	33334964	82548501	185179442	82548926	82548588	-	82549090
Brasiliscincus caissara	33334946	33334963	-	-	-	-	-	-
Brasiliscincus heathi	82548852	33334961	82548489	82548771	82548970	82548609	-	82549170
Caledoniscincus	190147915	190147906	334903284	190147817	-	-	-	-
Calyptotis	30691165	34821427	-	24110612	-	-	-	-
Carlia	30691152	56567390	61102768	5881289	-	85692054	-	-
Celatiscincus	-	-	113015800	-	-	-	-	-
Chalcides	55831607	410111711	410111633	238867264	-	-	410111646	-
Chioninia coctei	22657712	-	-	18000927	-	-	-	-
Chioninia delalandii	27447024	27447041	13446889	13624817	-	-	-	-
Chioninia fogoensis	22657706	37722632	13446893	18000906	-	-	-	-
Chioninia nicolauensis	-	-	-	18000911	-	-	-	-
Chioninia spinalis	27447007	27447028	13446895	13624793	-	-	-	-
Chioninia stangeri	22657695	37722630	13446901	13624799	-	-	-	-
Chioninia vaillantii	22657726	37722634	13446899	18000964	-	-	-	-
Coeranoscincus	30691166	30691206	-	-	-	-	-	-
Coggeria	115501531	115501555	-	-	-	-	-	-

Copeoglossum aurae	387860912	387860931	-	341957017	-	-	-	-
Copeoglossum nigropunctatum	27447014	33334958	-	185179474	-	-	635718039	-
Corucia	34821625	34821431	334903286	-	-	-	-	-
Cryptoblepharus	34821626	34821432	113015802	61102847	-	-	-	-
Ctenotus	30691167	34821434	61102778	258677519	-	345450830	-	-
Cyclodomorphus	34821629	34821435	334903288	18000622	-	-	-	-
Dasia grisea	326652824	326652947	-	-	-	-	685806315	-
Dasia haliana	406356162	406356157	-	-	-	-	-	-
Dasia johnsinghi	406356164	406356159	-	-	-	-	-	-
Dasia olivacea	406356165	406356160	-	-	-	-	-	-
Dasia subcaerulea	406356161	406356156	-	-	-	-	-	-
Dasia vittata	8918288	8918299	-	-	-	-	-	-
Egernia	30691153	30691193	334903290	18000624	-	-	-	-
Emoia	33385532	9712366	33385290	1508798	33385418	-	326653533	-
Eremiascincus	30691170	34821439	334903292	-	-	-	-	-
Eugongylus	33385535	9712368	334903294	61102853	33385408	-	392936179	-
Eulamprus	30691172	30691213	61102776	14599369	-	-	-	-
Eumeces	37812359	410111718	11596347	82494565	-	39545956	410111662	-
Eumecia anchietae	34821658	34821464	-	-	-	-	-	-
Eurylepis	410111730	410111719	410111637	410111749	-	-	410111660	-
Eutropis beddomei	380504933	380504928	380504905	-	-	-	685806325	-
Eutropis bibronii	380504942	380504926	380504907	-	-	-	685806327	-
Eutropis bontocensis	-	-	-	-	-	-	685806331	-
Eutropis carinata	37724216	37724266	380504895	-	-	-	685806337	-
Eutropis clivicola	380504941	380504919	380504903	-	-	-	685806347	-
Eutropis cumingi	82548838	82548355	82548465	82548705	82548948	-	685806357	82549110
Eutropis dissimilis	37724218	37724268	-	-	-	-	-	-
Eutropis grandis	-	-	-	-	-	-	685806485	-
Eutropis indeprensa	37724219	37724269	-	-	-	-	685806375	-
Eutropis longicaudata	27447021	82548357	171345046	345523773	-	-	685806399	82549114

Eutropis macularia	440495506	18147747	82548463	82548703	82548946	82548598	685806405	82549108
Eutropis madaraszi	37724223	37724273	-	-	-	-	685806495	-
Eutropis multicarinata	37724224	37724274	113015770	-	-	-	685806439	-
Eutropis multifasciata	37724228	37724281	42521179	82548707	82548950	-	326653541	82549112
Eutropis nagarjunensis	380504935	380504915	380504911	-	-	-	685806453	-
Eutropis quadricarinata	37724232	37724282	-	-	-	-	685806457	-
Eutropis rudis	82548836	82548353	82548461	82548701	82548944	82548597	685806459	82549106
Eutropis rugifera	37724233	37724286	-	-	-	-	685806343	-
Eutropis trivittata	380504934	380504914	380504909	-	-	-	685806475	-
Eutropis tytleri	37724217	37724267	-	-	-	-	-	-
Exila nigropalmata	187763970	-	-	187763973	-	-	-	-
Feylinia	83853974	34821466	33385260	83853958	33385396	-	392936195	-
Glaphyromorphus	321271331	115501560	-	-	-	-	-	-
Gongylomorphus	55831623	34821469	61102792	225728549	-	-	-	-
Graciliscincus	-	-	113015764	-	-	-	-	-
Hakaria	55831624	34821537	61102808	238867272	-	-	-	-
Hemiergis	321271315	34821470	334903296	-	-	-	-	-
Hemisphaeriodon	34821665	34821471	-	-	-	-	-	-
Heremites auratus	391324541	37724263	-	37722676	-	-	-	-
Heremites septemtaeniatus	37724234	-	-	-	-	-	-	-
Heremites vittatus	391324552	37724291	-	185179522	-	-	-	-
Insulasaurus	-	-	-	-	-	-	326653555	-
Isopachys	9712344	9712370	-	-	-	-	-	-
Janetaescincus	55831625	55831666	61102800	238867268	-	-	319878875	-
Kanakysaurus	-	-	113015680	-	-	-	-	-
Lacertaspis	34821677	34821482	-	-	-	-	-	-
Lacertoides	-	-	113015684	-	-	-	-	-
Lamprolepis	33385531	33385481	334903298	33386012	321494400	82548576	-	82549068
Lampropholis	190148004	56567391	113015686	61102875	298349063	41584461	298349022	-
Lankascincus	34821681	34821486	-	-	-	-	-	-

Larutia	-	-	-	-	321494405	-	-	-
Leiolopisma	34821682	34821487	113015774	61102857	-	-	-	-
Lepidophyma	297185185	33385518	33385342	166077463	297185414	297185310	-	-
Lepidothyris	30691157	34821512	-	-	-	-	-	-
Leptosiaphos	34821690	34821490	158121088	-	-	-	-	-
Liopholis	-	-	334903302	-	-	-	-	-
Lioscincus	194396027	194396022	113015692	194396016	-	-	-	-
Lipinia	326652838	9712372	334903304	-	321494399	-	326653557	-
Lissolepis	-	-	334903306	-	-	-	-	-
Lygisaurus	33385538	33385488	33385282	33386026	33385412	-	-	-
Lygosoma	34821715	9712375	334903308	-	321494406	-	-	-
Mabuya berengerae	635717470	-	-	635172943	-	-	635718023	-
Mabuya dominicana	387860918	387860937	-	387860882	-	-	-	387860849
Mabuya mabouya	326652840	27447037	-	185179468	-	-	326653561	-
Mabuya meridensis	635717483	-	-	185179470	-	-	635718035	-
Mabuya pergravis	635717490	-	-	635172985	-	-	635718041	-
Mabuya zuliae	185536533	-	-	185179504	-	-	-	-
Madascincus	255766969	255766981	256009925	-	255766941	-	319878853	-
Manciola guaporicola	82548842	82548387	82548473	185179464	82549010	82548630	-	82549168
Marisora alliacea	185536525	-	-	-	-	-	-	-
Marisora aurulae	27447019	-	-	-	-	-	-	-
Marisora brachypoda	-	-	-	185179492	-	-	-	-
Marisora falconensis	635717479	-	-	185179454	-	-	635718029	-
Marisora roatanae	387860928	387860946	-	387860902	-	-	-	-
Marisora unimarginata	326652841	18147746	-	185179488	-	-	326653563	-
Marmorosphax	190147916	190147909	113015716	190147809	-	-	-	-
Melanoseps	83853978	33385497	33385300	83853964	33385424	-	319878881	-
Menetia	-	218158441	-	-	-	-	-	-
Mesoscincus	55831617	34821527	-	-	-	-	319878883	-
Mochlus	34821719	34821523	-	-	-	-	319878877	-

Morethia	30691160	190147910	334903310	190147807	-	-	-	-
Nangura	30691181	323146364	334903312	-	-	-	-	-
Nannoscincus	190147917	190147908	113015720	190147811	-	-	-	-
Niveoscincus	190148003	34821528	334903314	61102871	-	-	-	-
Notomabuya frenata	82548829	82548391	82548521	185179460	82548930	82548590	-	82549092
Notoscincus	30691182	34821529	334903316	-	-	-	-	-
Oligosoma	52630409	34821530	334903318	61102867	194396044	-	-	-
Ophiomorus	55831629	55831670	-	157502921	-	-	319878885	-
Ophioscincus	115501540	30691223	-	-	-	-	-	-
Orosaura nebulosylvestris	185536540	-	-	185179510	-	-	-	-
Otosaurus	-	-	-	-	-	-	-	326653565
Pamelaescincus	55831630	34821531	61102802	18000620	-	-	-	-
Panaspis	326652844	34821534	-	-	-	-	326653569	-
Panopa carvalhoi	82548887	82548404	82548561	185179450	82549044	82548644	-	82549196
Panopa croizati	185536514	-	-	185179452	-	-	-	-
Papuascincus	115501533	115501557	-	-	-	-	326653573	-
Paracontias	38884838	255766984	38884952	38884907	40068173	39545971	319878887	-
Parvoscincus	326652871	326652999	-	-	-	-	326653621	-
Phoboscincus	-	-	113015736	-	-	-	-	-
Pinoyscincus	-	-	-	-	-	-	326653677	-
Plestiodon	336359703	34821460	334903320	166077339	321494402	-	75993593	82549202
Prasinohaema	30691184	30691224	334903322	-	-	-	-	-
Proablepharus	-	-	334903324	-	-	-	-	-
Proscelotes	33385556	37723244	33385320	33386064	33385442	-	-	-
Pseudemoia	194396028	194396024	-	194396020	-	-	-	-
Pseudoacontias	37812364	37812318	-	-	-	-	-	-
Psychosaura agmosticha	82548834	82548351	82548457	82548699	82548940	82548595	-	82549104
Psychosaura macrorhyncha	82548833	33334957	82548455	82548695	82548938	82548623	-	82549160
Pygomeles	37812366	38884870	256009935	38884915	255766947	-	-	-
Ristella	34821734	34821538	-	-	-	-	-	-

Saiphos	30691185	34821539	-	24110636	-	-	-	-
Saproscincus	194396029	56567411	149929157	194396018	-	41584459	-	-
Scelotes	33385554	33385512	33385326	346986931	33385436	39545976	-	-
Scincella	321271332	34821546	334903328	283443082	321494404	-	326653719	-
Scincopus	55831634	410111716	410111635	410111743	-	-	410111658	-
Scincus	33385549	18147743	33385306	18000626	33385430	-	319879033	-
Sepsina	33385548	33385499	33385304	33386048	33385428	-	-	-
Sigaloseps	-	-	113015740	-	-	-	-	-
Simiscincus	-	-	113015760	-	-	-	-	-
Sphenomorphus	30691187	9712382	334903332	166077355	-	-	392936183	-
Spondylurus caicosae	387860914	387860934	-	387860876	-	-	-	-
Spondylurus culebrae	387860917	387860936	-	387860880	-	-	-	-
Spondylurus fulgidus	387860919	387860938	-	387860884	-	-	-	387860855
Spondylurus lineolatus	387860922	387860941	-	387860890	-	-	-	387860857
Spondylurus macleani	387860923	387860942	-	387860892	-	-	-	387860859
Spondylurus monitae	387860924	387860943	-	387860894	-	-	-	-
Spondylurus powelli	387860927	387860944	-	387860898	-	-	-	387860864
Spondylurus semitaeniatus	387860929	387860947	-	387860904	-	-	-	-
Spondylurus sloanii	185536523	387860949	-	185179482	-	-	-	-
Tiliqua	33385539	18147744	7330371	33386028	33385414	82548616	392936217	82549148
Toenayar novemcarinata	-	-	-	-	-	-	685806455	-
Trachylepis acutilabris	82548882	82548399	82548551	82548793	82549034	-	-	82549186
Trachylepis affinis	9712349	37724335	-	8885826	-	-	-	-
Trachylepis albilabris	37724295	18147749	-	-	-	-	-	-
Trachylepis atlantica	82548855	82548372	82548495	82548737	82548982	82548612	-	82549140
Trachylepis aureopunctata	440495514	18147757	82548429	82548669	82548912	82548581	-	82549076
Trachylepis binotata	440495487	8885801	440495408	-	-	-	-	-
Trachylepis boettgeri	440495491	27447035	82548431	82548671	82548914	82548582	-	82549078
Trachylepis brevicollis	62637842	62637844	-	62637847	-	-	-	-
Trachylepis capensis	82548879	82548396	82548545	82548787	82549028	82548639	-	82549182

Trachylepis comorensis	440495497	8885804	440495422	301032911	-	-	-	-
Trachylepis cristinae	391324544	391324557	-	-	-	-	-	-
Trachylepis dumasi	440495511	82548339	82548433	82548673	82548916	82548583	-	82549080
Trachylepis elegans	440495479	18147748	82548435	82548675	82548918	82548584	-	82549082
Trachylepis gravenhorstii	440495466	82548341	82548437	82548677	82548920	82548585	-	82549084
Trachylepis hoeschi	33385537	33385487	33385280	33386024	82548976	82548611	-	82549138
Trachylepis homalocephala	82548817	82548334	82548423	82548663	82548906	82548578	-	82549072
Trachylepis infralineata	-	-	-	301032961	-	-	-	-
Trachylepis irregularis	27447009	8885810	-	-	-	-	-	-
Trachylepis maculilabris	440495500	18147750	440495426	18000838	-	-	-	-
Trachylepis madagascariensis	440495480	82548342	82548439	82548679	82548922	82548586	-	82549086
Trachylepis margaritifera	22657666	37722624	-	18000834	-	-	-	-
Trachylepis nancycoutuae	440495470	-	-	-	-	-	-	-
Trachylepis occidentalis	82548883	323134846	82548553	82548795	82549036	-	-	82549188
Trachylepis ozorii	-	-	-	301032999	-	-	-	-
Trachylepis perrotetii	326652937	18147754	82548479	82548719	82548956	82548600	326653761	82549124
Trachylepis polytropis	18147738	18147753	-	-	-	-	-	-
Trachylepis punctatissima	-	-	82795112	82795216	-	-	-	-
Trachylepis quinquetaeniata	82548841	82548365	82548555	185179524	82549038	82548577	392936229	82549128
Trachylepis sechellensis	22657664	-	61102772	18000632	-	-	-	-
Trachylepis socotrana	391324546	391324559	-	18000842	-	-	-	-
Trachylepis spilogaster	33385533	33385483	33385272	33386016	33385404	39545955	-	-
Trachylepis striata	33385540	33385490	33385286	33386030	33385416	82548615	-	82549146
Trachylepis sulcata	440495485	8885822	440495406	37722646	-	-	-	-
Trachylepis tandrefana	440495462	-	440495370	-	-	-	-	-
Trachylepis tavaratra	440495518	-	440495458	-	-	-	-	-
Trachylepis tessellata	391324549	391324562	-	-	-	-	-	-
Trachylepis varia	440495493	8885823	440495418	301032989	-	-	-	-
Trachylepis variegata	82548880	82548397	82548547	82548789	82549030	82548640	-	82549184
Trachylepis vato	440495464	37724290	82548441	82548681	82548924	82548587	-	82549088

Trachylepis vezo	440495517	-	-	-	-	-	-	-
Trachylepis wrightii	440495499	37722623	61102770	18000634	-	-	-	-
Tribolonotus	30691162	34821567	334903336	300430396	-	-	-	-
Tropidophorus	34821765	9712385	113015776	-	-	-	-	-
Tropidoscincus	-	-	113015748	-	-	-	-	-
Typhlacontias	83853976	33385501	33385302	83853962	33385432	-	-	-
Typhlosaurus	33385522	323135036	33385250	323134931	-	82548568	319879037	82549052
Tytthoscincus	-	-	-	-	-	-	326653773	-
Varzea altamazonica	635717462	-	-	187763971	-	-	635718019	-
Varzea bistriata	635717472	387860932	82548449	185179448	82548932	82548643	635718027	82549062
Voeltzkowia	38884840	37812321	38884954	38884913	40068175	39545974	319879039	-
Xantusia	33385566	33385517	161177426	163890085	297185444	321440652	-	-
