# Supplementary figures and images for: Arrival and diversification of mabuyine skinks (Squamata: Scincidae) in the Neotropics based on a fossil-calibrated timetree
Source: PeerJ. 2017 Apr 18;5:e3194. doi: 10.7717/peerj.3194 (PMC5398276; doi:10.7717/peerj.3194)

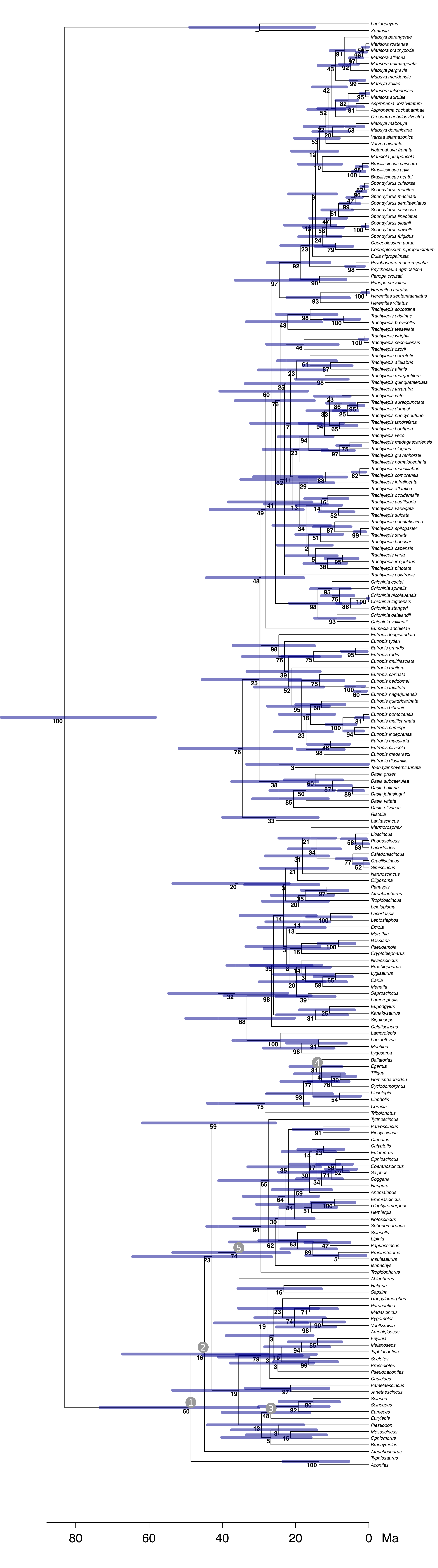

Supplement: Figure S1 — Maximum likelihood topology with divergence times inferred in MCMCTREE. Bars represent the 95% credibility intervals of node ages. Nodes on which calibration information was entered are indicated with numbered gray circles. Numbers in circles designate the following calibrations: (1) root (83.5–70.6 Ma); (2) Scincidae stem node (>20.4 Ma); (3) Eumeces (>13.6 Ma); (4) Egernia (>5 Ma) and (5) Tropidophorus (>13.6 Ma). [file peerj-05-3194-s004.png]
